# Hepatitis C Virus Infection Induces Autophagy as a Prosurvival Mechanism to Alleviate Hepatic ER-Stress Response

**DOI:** 10.3390/v8050150

**Published:** 2016-05-23

**Authors:** Srikanta Dash, Srinivas Chava, Yucel Aydin, Partha K. Chandra, Pauline Ferraris, Weina Chen, Luis A. Balart, Tong Wu, Robert F. Garry

**Affiliations:** 1 Department of Pathology and Laboratory Medicine, Tulane University Health Sciences Center, New Orleans, LA-70112, USA; schava@tulane.edu (S.C.); pchandr1@tulane.edu (P.K.C.); pferrari@tulane.edu (P.F.); wchen5@tulane.edu (W.C.); twu@tulane.edu (T.W.); 2 Gastroenterology and Hepatology, Tulane University Health Sciences Center, 1430 Tulane Avenue, New Orleans, LA-70112, USA; yaydin@tulane.edu (Y.A.); lbalart@tulane.edu (L.A.B.); 3 Microbiology and Immunology, Tulane University Health Sciences Center, 1430 Tulane Avenue, New Orleans, LA-70112, USA; rfgarry@tulane.edu

**Keywords:** Hepatitis C virus (HCV), Endoplasmic reticulum stress (ER-stress), Unfolded protein response (UPR), Autophagy, Chaperon-mediated autophagy (CMA), Interferon (IFN), Interferon-alpha receptor-1 (IFNAR1), chronic liver disease (CLD), Hepatocellular carcinoma (HCC)

## Abstract

Hepatitis C virus (HCV) infection frequently leads to chronic liver disease, liver cirrhosis and hepatocellular carcinoma (HCC). The molecular mechanisms by which HCV infection leads to chronic liver disease and HCC are not well understood. The infection cycle of HCV is initiated by the attachment and entry of virus particles into a hepatocyte. Replication of the HCV genome inside hepatocytes leads to accumulation of large amounts of viral proteins and RNA replication intermediates in the endoplasmic reticulum (ER), resulting in production of thousands of new virus particles. HCV-infected hepatocytes mount a substantial stress response. How the infected hepatocyte integrates the viral-induced stress response with chronic infection is unknown. The unfolded protein response (UPR), an ER-associated cellular transcriptional response, is activated in HCV infected hepatocytes. Over the past several years, research performed by a number of laboratories, including ours, has shown that HCV induced UPR robustly activates autophagy to sustain viral replication in the infected hepatocyte. Induction of the cellular autophagy response is required to improve survival of infected cells by inhibition of cellular apoptosis. The autophagy response also inhibits the cellular innate antiviral program that usually inhibits HCV replication. In this review, we discuss the physiological implications of the HCV-induced chronic ER-stress response in the liver disease progression.

## 1. Introduction to HCV Infection and Liver Disease

Hepatitis C virus (HCV) is a blood-borne pathogen that specifically infects the liver. HCV infection is prevalent worldwide, thus representing a major public health problem [1,2]. At present, there are 185 million people, representing 3% of the total world population, that are chronically infected with HCV [3]. Most individuals infected with HCV fail to clear the infection naturally, leading to a stage of chronic infection. Chronic HCV infection can progress to liver cirrhosis and hepatocellular carcinoma (HCC) [4]. HCV infection is associated with 15- to 20-fold increased risk of developing HCC [5]. Currently, there is no vaccine available to immunize people to prevent new HCV infection. Interferon-alpha (IFN-α) plus ribavirin (RBV)-based combination antiviral therapy has been used as a standard of care for patients with chronic HCV infection for the last ten years. However, this drug combination cleared infection in only 50% of patients [6].

**Figure 1:**
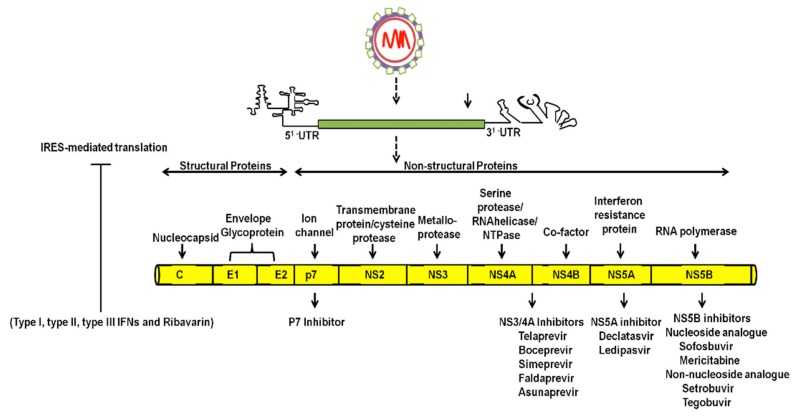
Structure of hepatitis C virus (HCV) RNA genome and ten different mature proteins are produced from the single large open reading frame (ORF). Type I, Type II, and Type III interferons, and ribavirin specifically inhibit the internal ribosome entry site (IRES, 5’UTR) function. Core, E1 and E2 are structural proteins and NS2–NS5 are non-structural proteins. The non-structural proteins (NS3/4A, NS5A and NS5B) are the targets of antiviral drug discovery. The 3’-UTR is important for HCV genome replication.

The recent development of potent direct-acting antivirals (DAAs) targeting the NS3/4A protease inhibitors, NS5B polymerase inhibitors and NS5A complex inhibitors, is changing the therapeutic options for curing HCV by antiviral therapy [7] (Figure 1). Significantly higher, sustained virological response rates (greater than 90%) have been achieved using the new direct-acting antivirals (DAAs), suggesting that these new antiviral therapies yield a high rate of viral clearance and reduce the associated risk of HCC development [8,9]. Additional versions of highly effective DAA combination treatments provide hope that HCV infection can be globally eliminated. This will require that all infected patients receive early diagnosis and access to antiviral treatment. Despite the success of new HCV treatments, understanding the mechanisms for how HCV counteracts host cell antiviral defense mechanisms to establish long-standing chronic liver disease that often progresses to liver cirrhosis and HCC remains an important area of future research. Elucidating the mechanisms of hepatocarcinogenesis induced by HCV will be of great interest to hepatologists since non-infectious metabolic conditions such as alcoholism and non-alcoholic steatohepatitis (NASH) also expose humans to an increased risk of liver disease, including cirrhosis and HCC [10].

Hepatitis C virus is an enveloped positive-stranded RNA virus belonging to the *Flaviviridae* family. HCV strains are classified into seven genotypes and many more subtypes based on variations in the nucleotide sequences of whole viral genomes [3]. HCV infection is initiated by the attachment and entry of the virus particles into hepatocytes by receptor mediated endocytosis. Multiple host cell surface proteins are required for attachment and entry cycle of the HCV particle into the hepatocyte through the clathrin-mediated endocytosis [11]. During the viral entry process, the fusion reaction of viral envelope with host endosomal membrane at low pH allows the release of free HCV genomic RNA into the cytoplasm. The positive strand RNA genome directly binds to ribosomes through the 5’ untranslated region (UTR) using an internal ribosome entry site (IRES) mechanism [12]. Translation of HCV genome leads to the production of a single large polyprotein of 3000 amino acids, which is subsequently cleaved by cellular and viral proteases into structural proteins, core, envelopes (E1 and E2) and non-structural (NS) proteins P7, NS2, NS3, NS4A, NS4B, NS5A and NS5B [13]. The structural proteins, core, E1 and E2 are necessary for formation of infectious virus particles and release, whereas the non-structural proteins are needed for HCV RNA replication [14].

**Figure 2:**
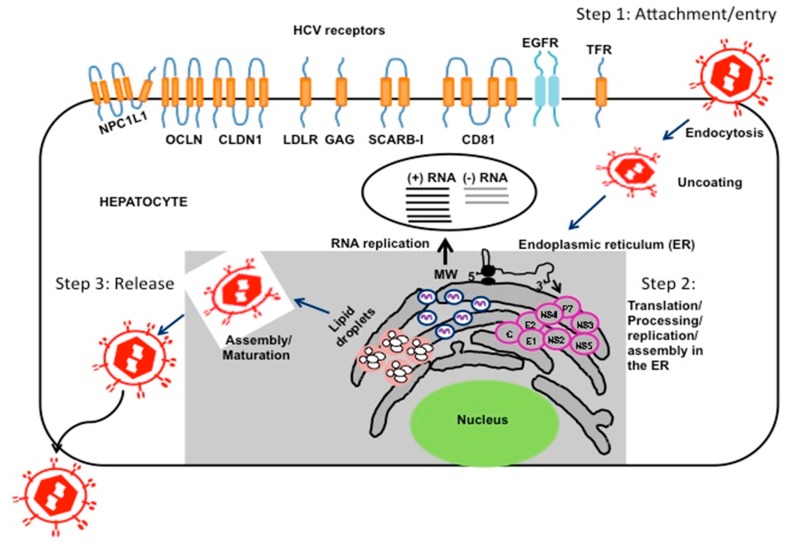
Infection cycle of HCV. The infection of HCV is initiated by the attachment and entry of virus particle through a number of cell surface receptors. The HCV RNA binds to ribosome and translates a single large polyprotein, which is a process into structural and non-structural proteins. Accumulation of viral proteins induces endoplasmic reticulum (ER)-stress response that leads to proliferation of ER-membranes and formation of membranous web structure. HCV replication produces many new genomic positive strand and negative strand RNA. Genomic HCV positive strand RNA packages into complete infectious virus particles that release through secretory pathway.

HCV is an RNA virus that replicates exclusively in the cytoplasm, without integration into the host cell genome. Sustained viral replication in hepatocytes leads to increased production of genome-length positive strand RNA, replicative intermediates (negative strand RNA) and viral proteins in the endoplasmic reticulum (ER). Replication of HCV in hepatocytes leads to proliferation and remodeling of the ER membranes into a structure referred to as the membranous web (Figure 2). Intrahepatic HCV replication stimulates lipid metabolism, leading to accumulation of lipid droplets in the multilayer membrane vesicles that facilitate virus assembly and maturation processes [15]. These ER-derived membranous vesicles support HCV replication and production of progeny viral RNA. Virus particle assembly and release occurs in the membranous web and is closely linked to very low-density lipoprotein (VLDL) synthesis and secretion [16]. HCV RNA replication is catalyzed by the viral RNA-dependent RNA polymerase (RdRp) NS5B leading to the production of negative strand RNA. Additional details on the precise mechanism of HCV replication, assembly and secretion have been summarized in several reviews [17,18,19,20].

After initial infection, HCV replication in hepatocytes, the predominant cell type in the liver, produces millions of new virus particles. In chronic HCV infection, about 10^12^ HCV virions are secreted into circulation every 24 h [15]. The continuous cycle of intracellular virus replication, virus release and re-infection is central to mechanisms of HCV persistence and pathogenesis. HCV also develops chronic infection in most infected individuals, suggesting that HCV infection promotes cell survival after the stress response has been triggered. The molecular mechanisms by which HCV develops persistent infection in the hepatocytes by maintaining a delicate balance between viral induced stress response and cell survival is not well understood. This is an important area of research that may increase our understanding of the interplay between long-lasting chronic HCV infection and development of hepatocellular cancer. Therefore, the purpose of this review is to provide an updated literature that will increase our understanding on the relationship among virus infection, ER-stress and autophagy. Then, we will highlight our current understanding of how HCV attenuates this ER-stress response through autophagy induction. Finally, we discuss how the ER-stress, autophagy and inflammation are involved in the progression of liver disease in hepatitis C virus infection.

## 2. Relationship among Virus Infection, ER-Stress Response and Autophagy

The endoplasmic reticulum (ER) and Golgi apparatus consists of an extensive membrane network that stretches from cell nucleus to plasma membrane in all mammalian cells. The membrane bound compartment of ER/Golgi is connected to most of the organelles through tubules and vesicles [21]. The ER and Golgi network is vital to the cellular biosynthesis processes. The ER has multiple functions inside the cell including calcium storage, synthesis of secreted and resident proteins, protein folding, lipid metabolisms, carbohydrate synthesis and drug-detoxification [22]. Secretory and membrane bound proteins are synthesized on ribosomes and translocate to the ER-lumen undergo folding, organelle-specific post-translational modification in order to assemble into a higher-order structure. A number of protein chaperons are present in the ER lumen to facilitate protein folding, disulfide bond formation and carbohydrate modifications. The ER-lumen is rich in calcium-dependent molecular chaperones such as ER-luminal binding proteins (Bip), calmodulin (CAM), and calreticulin (CRT), which assist in protein folding [23]. The ER-lumen also has an oxidative environment, which is essential for protein disulfide isomerase (PDI)-mediated disulfide bond formation needed for protein folding [24]. The ER-homeostasis is perturbed during virus infection because the infecting virus produces large amounts of viral proteins. When the viral protein load in the lumen exceeds the capacity of ER, this leads to stress response called ER-stress. Many non-viral insults such as chemical, drug accumulation, energy deletion (ATP) and nutrient deprivation have been shown to induce ER-stress. All the viral and non-viral insults lead to imbalance in cellular redox equilibrium, calcium homeostasis, post-translational modification and an increase in protein synthesis [24]. The ER-stress response can be acute or chronic. To handle the stress response, mammalian cells generate a cytoprotective gene transcription cascade in the nucleus called the unfolded protein response (UPR) [25]. The purpose of UPR is to reduce the stress response by enhancing protein folding, decrease the protein load, and expansion and rearrangement of ER-membrane. In addition, the UPR alters multiple cellular processes such as autophagy, apoptosis, ER/Golgi biosynthesis and lipid synthesis. If the stress is severe, such that the ER-stress cannot be resolved despite these adaptive measures, then this can lead to a programmed cell death (apoptosis). Excess ER-stress leads to UPR mediated cell death through activation of caspase-12 [25]. Some examples of acute stress response such as hypoxia, ischemia, calcium depletion, and glucose depletion can be tolerated due to rapid induction of UPR genes [26,27]. During the acute stress the expression of UPR increased but their expression levels returned to base line levels after some time. The purpose of the induced UPR is to restore the ER-function and relieve the stress response.

During the viral infection, the expression of UPR genes does not return to base line expression levels, rather, it remain elevated throughout the infection, leading to chronic ER-stress. This is because many positive strand RNA viruses, including HCV, house the virus replication machinery in the protective ER-membrane. Viruses need host ER to produce increased quantities of viral proteins to continue replication. To continue the replication cycle, many viruses have evolved strategies to manipulate the host UPR in various ways to stimulate protein synthesis capacity, increased degradation of misfolded proteins and to improve cell survival by inhibiting cellular apoptosis. The UPR eliminates cytotoxic misfolded proteins by ubiquitination (Ub) and proteasome-mediated degradation, which is called ER-assisted degradation (ERAD). Accumulating evidence now suggests that virus associated ER-stress and UPR induces cellular autophagy as a major protein degradation mechanism to alleviate stress response and cell survival. The extent of UPR activation during acute and chronic infection varies among different viruses [28]. For example, some viruses induce part of the UPR by direct interaction with Bip, as seen with the US11 protein of human cytomegalovirus (HCMV) [29]. Some examples including human cytomegalovirus induce X-box binding protein 1 (XBP1) splicing but do not induce activating transcription factor 6 (ATF6) cleavage [30], West Nile virus activates XBP1 splicing and induces ATF6 but inhibits protein kinase RNA (PKR)-like ER kinase (PERK) activity [31], lymphocytic choriomeningitis virus activates ATF6 but not PERK or inositol-requiring protein-1 (IRE1) [32], EBV appears to activate all three axes [33], and herpes simplex virus (HSV) activates DNA damage-inducible protein 34 (GADD34) target to relieve translational inhibition [34]. Many researchers have verified that the induction of UPR is essential for promoting viral replication in the ER and inhibition of UPR pathways stops virus replication and increase cellular apoptosis.

The viral related UPR triggers host inflammatory signaling cascade through mitogen-activated protein kinases (MAPKs), c-Jun N-terminal kinase (JNK), and p38 kinases that activate the nuclear factor-kB (NF-kB) as a result of chronic ER-stress [26,27]. Increasing evidence supports that the UPR signaling synergistically interacts with viral innate immune sensing signaling to produce inflammatory cytokines and type I IFN, which are vital for innate and adaptive immune responses during chronic virus infection. Recent studies suggest that ER-stress is a hallmark of many common diseases such as inflammation, viral infection, cancer and many metabolic diseases. Induction of ER-stress and UPR has been verified in acute and chronic liver diseases. The molecular details for how ER-stress induces such autophagy response that triggers liver disease progression is an active area of future investigation. In the following section, we will review the work of many researchers, including our own, regarding how the HCV infection and other non-viral insults induce ER-stress and UPR in chronic liver diseases.

## 3. Hepatitis C Virus Infection Induces Chronic ER-Stress and UPR

Hepatocytes are responsible for a myriad of metabolic processes, such as protein synthesis and metabolism of lipids and carbohydrates [35]. HCV causes massive rearrangements of intracellular membranes in the ER that serve as sites for viral replication and protein accumulation [36]. As noted, the ER responds to accumulation of unfolded viral proteins in its lumen (ER-stress) by activation of intracellular signal transduction pathways called the UPR to alleviate the ER-stress response [37,38,39] The molecular interactions for how infected hepatocytes respond to this stress and integrate the stress response to improve cell survival are not well understood. In general, hepatocytes cope with the ER stress in four different steps. First, infected cells attenuate translation to reduce the protein load in the ER, prior to mRNA encoding of UPR-proteins. Second, the UPR improves ER function through inducing chaperone gene expression. Third, proliferation of ER compartments accommodates the viral protein load, initiating ER-associated degradation (ERAD) of unfolded proteins, using ubiquitin–proteasome and autophagy pathways. Fourth, if the ERAD response fails to resolve the ER stress, then the UPR switches from pro-survival signaling to apoptosis signaling. In addition, the ER-stress and UPR are activated in several metabolic syndromes such as obesity and type II diabetes, both of which contribute synergistically to the pathogenesis of liver disease [40]. It is unclear whether the chronic ER-stress or the attenuated ER-stress response contributes to evolution of chronic liver disease and development of hepatocellular carcinoma.

**Figure 3:**
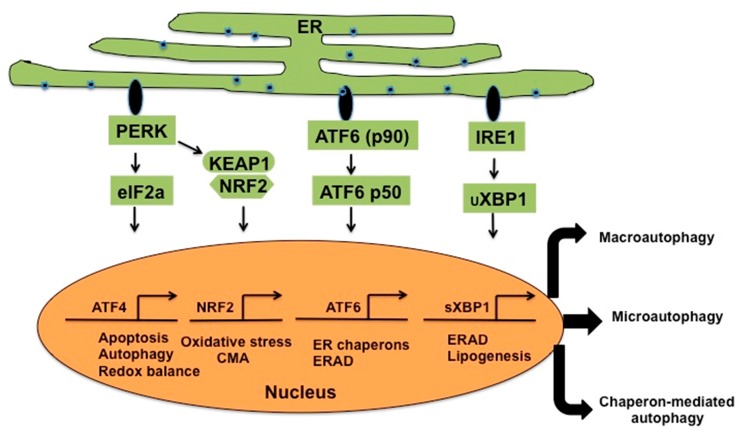
Schematic representation of unfolded protein response (UPR) associated with ER stress in HCV infection. The three ER stress sensors, protein kinase RNA (PKR)-like ER kinase (PERK), activating transcription factor 6 (ATF6) and inositol-requiring protein-1 (IRE1), activate gene expression that is involved in reducing ER-stress through a wide variety of mechanisms. PERK phosphorylates eukaryotic translation initiation factor 2A (eIF2a) to decrease translation while it activates activating transcription factor 4 (ATF4). ATF4 translocates into the nucleus to initiate transcription of genes involved in cellular apoptosis and autophagy. The ATF6 translocates to Golgi, where it is cleaved into an activate form. The active form of ATF6 translocates to the nucleus to activate genes involved in protein degradation and protein folding. The IRE1 decreases protein loads in the ER by enhancing mRNA degradation. The IRE1 axis also generates spliced versions of XBP1, which translocate to the nucleus to induce genes involved for protein degradation and lipogenesis.

In mammalian cells, UPR is activated by three classes of ER-stress sensors: PERK, ATF6, and IRE1 (Figure 3). When the accumulation of HCV proteins exceeds the folding capacity in the ER, this leads to the activation of ER-stress [40]. The ER luminal glucose-regulated protein (GRP78 is also known as BiP) are stress inducible molecular chaperones that regulate cell viability and apoptosis. In uninfected cells, GRP78 functions as UPR signaling by binding and inactivating three branches of ER-stress transducers (PERK; IRE1 and ATF6) [41]. When sufficient HCV protein accumulates in the ER, GRP78 is titrated away by binding to the misfolded viral proteins. This triggers the activation of PERK, IRE1 and ATF6 [42]. Activation of the UPR reduces ER-stress by attenuating translation and degradation of unfolded proteins, and increasing the size of the ER and its folding capacity.

In addition to reducing ER-stress, activation of each pathway also controls cell survival through induction of autophagy. The first ER stress sensor, PERK, phosphorylates eukaryotic initiation factor-2-alpha (eIF2-α) at Ser51, preventing the exchange of GDP and GTP, which effectively blocks the initiation of protein synthesis required for recovery from ER stress. However, activation of PERK favors increased translation of ATF4 under ER stress [43]; ATF4 is a master regulator that plays a crucial role in the cellular adaptation to the stress response by inducing transcription of numerous genes, including CCAAT/enhancer-binding protein, (C/EBP)-homologous protein (CHOP), and GADD34 [44]. CHOP, GADD34, and ATF4 trigger apoptosis upon prolonged activation of PERK [45]. The expression of ATF4 and CHOP drives the expression of autophagy genes *ATG5* and *LC3B*. PERK also phosphorylates the nuclear factor erythroid 2-related factor 2 (NRF2), which promotes transcription regulation of genes containing the antioxidant response elements (ARE) that are involved in protection against oxidative stress. The activation of the second stress sensor, ATF6, leads to its translocation from the ER to the Golgi apparatus, where the inactive form of 90-kDa ATF6 is cleaved to the active form of 50-kDa ATF6. Nuclear translocation of active form ATF6 then leads to transcription of chaperone genes such as GRP78 (BiP), GRP94, GRP170, GRP75 and other heat-shock protein families (e.g., HSC70) [46]. The ATF6 axis helps to increase ER-folding capacity during ER-stress [47]. The activation of the third sensor leads to phosphorylation of IRE1 and induces the RNase activity of IRE1. IRE1 removes a 26-nt intron from the mRNA, producing a stable form (sXBP-1) rather than the highly unstable unsliced XBP-1 (uXBP-1) protein [48]. The sXBP-1 is a potent transcription factor that enters the nucleus and induces transcription of the majority of UPR target genes [48]. In the liver, the XBP and CREBH (cAMP responsive element-binding protein, hepatocyte specific) pathways have been shown to regulate various aspects of hepatic lipid metabolisms. All three UPR axes are important in sustaining HCV replication and silencing each component has been shown to be detrimental to HCV RNA replication (reviewed in [49]).

Since HCV infection results in the production of large amounts of structural (Core-E1/E2/P7) and non-structural proteins (NS2-NS5B) in the ER, initial studies have been carried out to determine which viral protein contributes more to the ER-stress response. Results of these analyses suggest that retention of both structural proteins (E1 and E2) as well as non-structural proteins triggers ER-stress response (reviewed in [50]). The availability of the sub-genomic replicon based model system has allowed us to address the effect of HCV replication on ER-stress response. Replication of sub-genomic RNA has been found to induce ER-stress response and activation of UPR genes in Huh-7 cells (hepatoma cell line) [51,52]. The availability of the infectious HCV cell culture system has permitted an increased understanding of the effect of whole HCV genome replication on cellular ER-stress response. A number of laboratories have demonstrated that HCV infection of Huh-7 cells activates all three ER-stress sensors [53,54,55,56,57,58]. Merquiol and coworker [56] have measured the mRNA and protein levels of UPR genes in persistently HCV infected Huh-7.5.1 cells for up to 14 days. These investigators found that HCV-induced UPR gene expression (PERK, ATF6 and spliced XBP1) peaks on Day 3 and remains elevated until Day 14, compared to uninfected cultures. These results suggest that HCV infection perturbs ER homeostasis, causing chronic ER-stress. They confirmed that HCV infection induces adaptation to chronic ER-stress, since they did not find any increase in the expression of UPR gene induction in the HCV infected culture at Day 14 after thapsigargin (TG) treatment.

One of the limitations of HCV related ER-stress studies is that most of the investigations used Huh-7 cells, which are known to have intrinsically high ER-stress. Therefore, verification of HCV induced ER-stress response has been made by few researchers using more authentic replication models that use non-transformed infected primary human hepatocytes culture and humanized mice models. Importantly, increased expression of UPR genes (BiP and CHOP) has been shown using HCV infected human hepatocytes in SCID/Alb/uPA mice (chimeric mice with human livers) of HCV 1a H77 strain as well as HCV 2a JFH1 strain [58,59]. We have developed a robust HCV replication model in primary human hepatocytes (PHH) using human serum and showed that HCV replication can be maintained in PHHs for more than 15 days. In the infected PHH culture model, we demonstrated that HCV infection induced expression of three branches of UPR gene expression in infected primary human hepatocytes. The expression of UPR gene was minimal at Day 0 and HCV infection of PHHs induced UPR gene expression in a time-dependent manner examined up to 15 days, suggesting that HCV infection perturbs ER-homeostasis leading to a stage of chronic ER-stress response [60,61]. The UPR response did not decrease to the base line level, indicating that HCV infection of PHH induces chronic ER-stress.

Four separate studies have been performed to document the presence of HCV induced UPR gene expression using human liver tissues of patients who have been chronically infected with HCV [60,62,63,64]. Shuda and coworker [62] examined the expression of GRP78, ATF6 and XBP levels using surgically resected HCC and surrounding non-cancerous liver tissues from 13 patients infected with HBV or HCV. They found high expression of GRP78 in the cytoplasm, nuclear translocation of activated ATF6, and spliced version of XBP mRNA, indicating that the presence of chronic ER-stress response in advanced liver disease. Asselah and coworkers [63] investigated ER-stress and UPR expression in liver samples from 28 untreated chronic HCV patients, 13 with mild fibrosis and 15 with advanced fibrosis. They confirmed altered hepatocyte ER-morphology in livers from patients with hepatitis C compared to normal livers. The authors also showed the activation of UPR (ATF6, IRE1 and PERK) pathways in mild and advanced liver fibrosis, which is indicative of the presence of chronic ER-stress in advanced liver disease. Chandra and coworkers showed the presence of UPR gene expression (IRE1, Bip and peIF2-α (phosphorylated eukaryotic initiation factor 2 alpha) in advanced chronic HCV patients and liver cirrhosis patients with viral and non-viral etiology by Western blot analysis [60]. This investigation showed the increased expression of UPR genes in chronic HCV infection as well as in advanced liver disease, suggesting the presence of chronic ER-stress. However, McPherson and coworkers [64] investigated the role of UPR in the pathogenesis of chronic HCV infection using 124 consecutive liver biopsies by histological staining and real-time RT-PCR for mRNA levels of GRP78, GRP94, sXBP1, CHOP, and GADD34. They found no evidence of the UPR in patients with chronic HCV infection. Overall, chronic ER-stress related to HCV infection appears to be a key event that explains the mechanisms of viral persistence and viral carcinogenesis.

Many researchers have examined the contributions of ER-stress to the development of viral and non-viral related liver disease over the last two decades [reviewed in 65]. The ER-stress response and UPR gene expression has been demonstrated in liver disease associated with hepatitis B [66] and hepatitis C virus infection [67], alcoholic liver diseases [68], nonalcoholic steatohepatitis (NASH) [69,70], ischemia perfusion liver injury, [71] and cholestatic models of liver diseases [72]. One recent study found that PERK and IRE1 pathway is activated in chronic HBV infection, whereas the ATF6 pathway is activated more during acute on chronic liver failure [73]. It has been demonstrated that ER-stress induces hepatic steatosis and increases hepatic lipogenesis [69]. The UPR sensors, PERK and ATF4 signaling pathways are required for hepatic very-low-density-lipoprotein receptor (VLDLR) upregulation. These authors show that ER-stress dependent VLDLR expression in primary human hepatocyte induced triglyceride accumulation in the presence of low-density lipoprotein [70]. It has been found that ER-stress related to fatty liver disease induces inflammatory processes through the activation of NrF2, JNK and NF-kB pathways [74]. ER-stress induces lipid biosynthesis and autophagy is used to degrade intracellular lipid stores, a mechanism that has been verified by number of investigators [74]. A connection between autophagy and development of hepatic steatosis in HCV-induced liver disease has been made in a recent study published by Vascovo *et al.* [75]. These investigators examined the role of autophagy in lipid degradation in cells infected with the whole virus or replicons as well as liver biopsies from patients with HCV. They showed HCV induced autophagy response is needed for lipid degradation. They also showed inhibiting cholesterol biosynthesis by statins decreased autophagy and virus replication, suggesting that lipid metabolism is linked to UPR and autophagy. Hepatic ER-stress that promotes ballooning degeneration of hepatocytes is induced during alcoholic liver injury in human and mice [65]. Hepatitis B virus infection has also been shown to activate UPR response and induce expression of IRE-1/XBP1 and ATF6 pathways [66]. The ER-stress response is induced in acetaminophen drug induced liver injury [76]. Liver injury induced during ischemia perfusion has been demonstrated to induce oxidative stress, NF-kB activation, ATP depletion and calcium release from the ER [71]. Liver injury during cholestasis due to the accumulation of toxic bile salts has been reported to promote UPR and activate GRP78, CHOP and NF-kB pathways [72]. It is now becoming clear that ER-stress response seems central to the mechanisms of inflammatory liver disease, cell injury and steatosis. The molecular mechanism for how chronic ER-stress mediates the pathogenesis of liver disease remains elusive. Overall, these data suggest that HCV induced UPR activation is more prominent in advanced liver disease than chronic liver disease. It is also possible that chronic HCV infection can result in adaptive or chronic UPR response similar to non-alcoholic fatty liver diseases [77]. The mechanisms by which HCV infected cells survive under ER-stress are unknown. The relationship between ER-stress and autophagy in the evolution of chronic liver disease and cancer has become a hot spot for research. In the following section, we review the progress made during the last few years understanding the mechanisms of autophagy initiation related to ER-stress during virus infection.

## 4. Mechanisms of Autophagy Initiation

Autophagy is an intracellular degradation mechanism that every mammalian cell performs in order to survive under stress conditions related to virus infection or nutrition starvation, to promote cell survival [78]. The increased autophagy associated with ER-stress leads to cell death via apoptosis in some virus infections [79]. Other viruses such as dengue virus, human immunodeficiency virus (HIV) and hepatitis C virus, induce the cellular autophagy response to enhance viral replication and prolong the survival of virus-infected cells by counteracting the apoptotic response [80,81,82]. In addition, autophagy can be induced in virus infection as a mechanism of generating energy needed for biosynthesis of new macromolecules by recycling metabolites, such as amino acids, sugars and lipids, produced during lysosomal proteolysis [83,84,85]. The molecule AMP-kinase (AMPK) senses cellular energy requirements through AMP to ATP ratios in the cell cytoplasm. High AMP levels reflect low energy states in the cell, and under these conditions, AMPK can initiate autophagy through inactivation of mTOR1 (a mechanistic target of rapamycin complex 1) or by phosphorylation of ULK1/2 protein. Another autophagy-inducing signal is related to inhibition of mammalian target of rapamycin (mTOR1) by depletion of amino acid levels in the cytoplasm. The cellular autophagy response is increased as a result of shortage of nutrients (amino acids, sugar, lipids, nucleotides and ATP) required for sustaining HCV replication [86,87]. Decreasing mTOR1 levels caused by low nutrient levels activate autophagy signaling. The initiation and termination of autophagy are linked to cellular nutrient sensing mechanisms [84].

**Figure 4:**
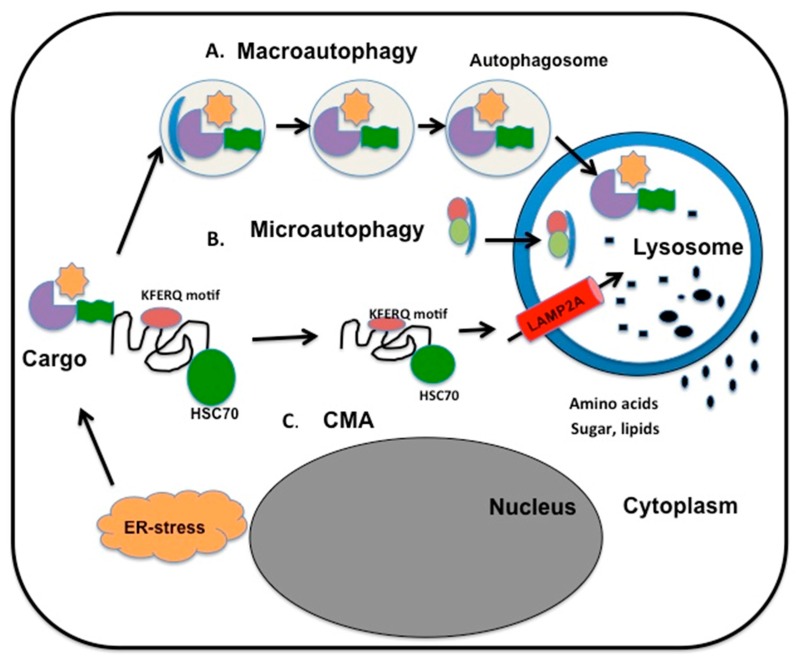
Schematic illustrations of different types of autophagy. (**A**) During macroautophagy, the cargo targeted for destruction is enclosed in double-membrane vesicles called autophagosomes that deliver the contents to the interior of lysosome through membrane fusion. The metabolites (amino acids, sugars and lipids) are then released into the cytoplasm for the synthesis of new macromolecules or as a source of energy. (**B**) In microautophagy, the cargo directly enters the lysosome by membrane invagination and degradation. (**C**) During chaperone-mediated autophagy (CMA), proteins with pentapeptide KFERQ-like sequences are recognized by the heat shock cognate protein complex 70 (HSC70) chaperone. This complex then binds to LAMP2A on the lysosome membrane for subsequent internalization and degradation.

Persistent HCV replication induces three types of autophagy in mammalian cells: macroautophagy, chaperone-mediated autophagy (CMA), and microautophagy [88,89,90,91]. The differences among these three types of autophagy are illustrated in Figure 4. There is evidence that ER-stress activates all three branches of autophagy machinery [92]. In macroautophagy, a portion of cytosol is engulfed by a double-membrane structure called the autophagosome, which fuses with a lysosome to become an autophagolysosome. The contents of autophagolysosomes are degraded by proteases, lipases, nucleases, and glycases. Several cellular components, including the endoplasmic reticulum (ER), Golgi/*trans*-Golgi apparatus, and plasma membrane, participate in autophagosome formation. In microautophagy, cytosolic material is directly engulfed by the lysosome via membrane rearrangement. Recently, microautophagy has been renamed on the basis of the cargo it degrades, as mitophagy, pexophagy, reticulophagy, and ribophagy. A recent study shows that HCV infection promotes mitochondria fission and mitophagy to attenuate cellular apoptosis and promote persistent viral replication [89]. Chaperone-mediated autophagy (CMA) is responsible for degradation of cytosolic proteins induced under oxidative stress conditions through the PERK and IRE1 axes of the UPR [90,91,92]. All CMA substrates contain a consensus pentapeptide motif (KFERQ) that is recognized by a cytosolic chaperone, for example, heat shock cognate protein complex 70 (HSC70) [93,94]; As shown in Figure 4, HSC70 binds to lysosome-associated membrane protein 2A (LAMP2A), which results in the direct translocation of unfolded protein substrate across lysosomal membranes and subsequent degradation of the cytosolic proteins.

**Figure 5:**
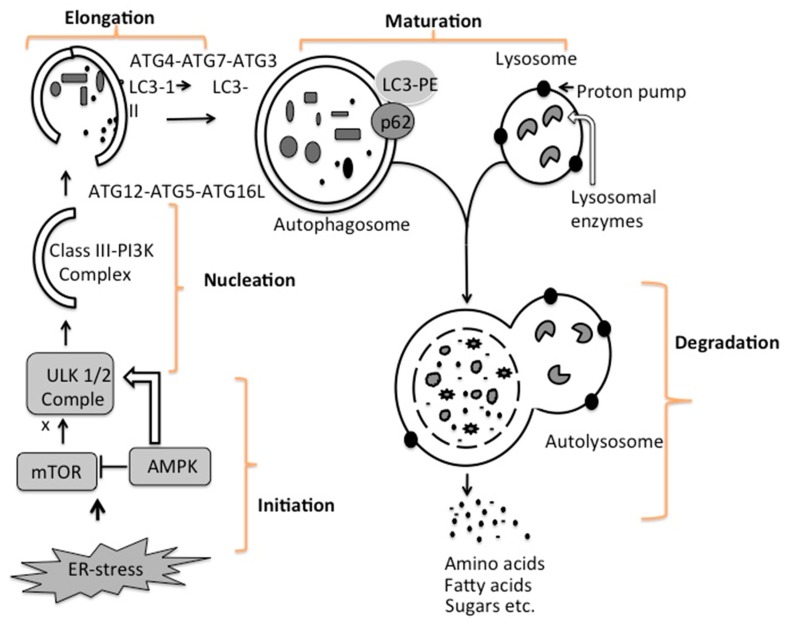
Molecular interactions involved in macroautophagy. Activation of unfolded protein response due to HCV replication in the ER initiates autophagy through direct or indirect activation of genes involved in autophagy. Autophagy responses also initiated due to low energy conditions by AMP-activated kinase (AMPK) and mechanistic target of rapamycin complex 1 (mTOR1). The phosphorylation of the ULK complex can be initiated by AMPK activation due to low ATP or mTOR inhibition due to low nutrients sensing. Activation of ULK complex initiates a series of reactions that leads to the engulfment of the cellular constituents in a double-membrane structure called autophagosome. The autophagosome then fuses with lysosome to form autophagolysosome in which the contents are digested by lysosomal enzymes into basic nutrients (sugars, lipids, amino acids and nucleosides), which are released into the cytoplasm for subsequent use. (Figure adapted from [102]).

In general, macroautophagy (autophagy) is coordinated at five different steps, known as initiation, nucleation, elongation, maturation, and degradation (Figure 5). During basal-level autophagy, Unc-51-like kinase (ULK1/2), ATG13, FIP200, and ATG101, exist in an inactive complex with mTOR1. The first step in the initiation of autophagy is the activation of the molecular complex of serine/threonine kinase ULK1. The activation of this ULK1 complex is usually suppressed by mTOR. A drop in energy status (amino acids, sugar and ATP), as well as ER-stress associated with virus infection, decreases the mTOR level, leading to activation of autophagy [95]. A decrease in mTOR activity increased phosphorylation and translocation of the ULK complex (ULK1–ATG13–FIP200–ATG101) from the cytoplasm to the ER [96,97,98]. The interaction of the ULK complex with ER-resident proteins leads to autophagy initiation [99]. The second step, nucleation, occurs as the ULK complex enlarges, due to interactions with the class III phosphatidylinositol 3-kinase (PI3K) complexes consisting of either Beclin 1–ATG14L–PI3KCIII–p150–Ambra1 or Beclin 1–UVRAG–PI3KCIII–p150–Bif1 [100]. Recruitment of additional ER-resident proteins such as double FYVE domain-containing protein 1 (DFCP1) and WD-repeat protein interacting with phosphoinositides (WIPI) facilitate the nucleation and creation of a curved double-membrane structure from the ER. This process, also called phagophores formation, usually starts with the isolation of a membrane fragment derived from the ER. The autophagy process can be inhibited at this step if Beclin 1 forms a complex with Rubicon or anti-apoptotic protein Bcl2 [101]. The third step is called elongation, when the two edges of phagophore membrane are extended by ubiquitin-like protein conjugation systems to sequester part of the cytoplasm. In this reaction, ATG12–ATG5 complex associates with ATG16 to form ATG12–ATG5–ATG16 (ATG16L), which localizes at the autophagosomal membrane. Another factor that is important for autophagy is the microtubule-associated protein light chain 3 (LC3), a cytosolic protein.

During autophagy, LC3 is covalently linked by ATG3, ATG4 and ATG7 to phosphatidylethanolamine (PE), a phospholipid for membrane insertion. This lipidation allows LC3 to localize to autophagosomal membrane. The LC3–PE is localized at the inner and outer membranes of the autophagosome. During elongation, the LC3 protein on the autophagosome can interact with misfolded and polyubiquitinated proteins that need to be degraded through autophagy such as p62 and NBR1 [103,104,105]. The fourth step, maturation, is related to autophagosome completion. The autophagosome undergoes two maturation steps. First, the autophagosome fuses with multivesicular endosomes to form an amphisome, the step where proton pumps are acquired for acidification. Second, the amphisomes fuse with a lysosome to become an autolysosome. Subsequently, the cellular materials present inside the autolysosome are degraded by the action of different lysosomal enzymes into amino acids, lipids, and sugars. The degradation products, such as amino acids, lipids, and sugars, are released from the autolysosome via lysosome efflux transporters for reuse, including production of new proteins. The release of nutrients from the autophagolysosome reactivates mTOR, which triggers autophagy termination and the formation of nascent lysosomes. This process is called autolysosome reformation (ALR). Under conditions of low nutrition, the cycle is repeated. It is now believed that inhibition of mTOR1 is due to low energy state in the cell activates autophagy, whereas activation of mTOR1 is due to a high-energy state inhibits cellular autophagy.

The mechanism for how autophagy is induced during virus infection is just beginning to be understood. Many positive-stranded RNA viruses replicate in the ER-membranes, causing new membrane synthesis and rearrangement through induction of UPR [106]. At present, large bodies of literatures show that the autophagy process is induced after infection with wide range of DNA and RNA viruses (reviewed nicely in [107]). For example, Wang *et al.* and coworkers [108] study shows that PERK eIF2-α ATF4 and ATF6 pathways promote autophagy induction after HCV infection of Huh-7.5 cells. HCV core protein also directly induces autophagy gene expression (ATG12 and LC3). Hepatitis B virus is another hepatotropic virus that infects hepatocytes and induced autophagy through ER-stress mechanism [109]. Only surface antigens induced autophagy through all three UPR axes, whereas X-protein induces autophagy through activation of P13K signaling. Vesicular stomatitis virus (VSV) induces autophagy through down-regulation of mTOR activity and inactivation of Akt signaling [110]. Lastly, DNA viruses such as varicella zoster virus (VZV) can also induce autophagy via ER-stress and induction of UPR [111]. However, the cause–effect relationship between ER-stress and autophagy initiation mechanisms is not well understood for most of the viruses. Understanding the autophagy induction through UPR is important since UPR inhibitor or autophagy inhibitors can be used to inhibit virus replication. In the following section, we review how HCV infection induces different forms of autophagy to survive under stress conditions.

## 5. Hepatitis C Virus Infection Promotes Macroautophagy, Mitophagy, and CMA

At present, several studies have shown that autophagy response is induced in primary human hepatocytes, transformed hepatocytes and hepatoma cell line (Huh-7) after HCV infection [112,113,114,115,116,117,118,119,120,121,122,123,124,125,126]. The verification of autophagy induction in HCV-infected cells was made by the measurement of LC3-II levels by Western blot analysis, clearance of p62 expression, and induced expression of ATG proteins by Western blot analysis. Autophagy induction after virus infection was confirmed by demonstration of autophagic vesicles (autophagosomes) by transmission electron microscopy. The induced autophagy response has been confirmed using liver biopsies of patients who are chronically infected with hepatitis C virus. Rautou and coworkers [121] compared the autophagy response in liver biopsies derived from chronic HCV patients by electron microscopy and Western blotting of LC3B. They demonstrated that the number of autophagic vesicles present in chronic HCV patients was six fold higher than the liver tissues from patients with chronic HBV infection, alcoholic and non-alcoholic steatohepatitis (NASH). Another study by Vescovo and coworkers [75] confirmed the presence of HCV induced autophagy response in patients with microvescicular steatosis by LC3 immunoblotting. In this study the HCV induced autophagy response inversely correlated with the presence of microvesicular steatosis. Studies performed in our laboratory [57,60] demonstrated the HCV induced autophagy response in infected PHHs. We also verified autophagy induction using liver tissue from humans chronically infected with HCV [60]. The cellular ER-stress and autophagy response was high in patients with chronic HCV infection, with or without liver cirrhosis as measured by Western blotting. Beclin expression was low in the liver cirrhosis as compared to chronic HCV infection. These results indicate that the autophagy response persists in chronic liver disease and liver cirrhosis due to HCV infection.

HCV infection has been shown to induce microautophagy that selectively removes or degrades mitochondria in the process called mitophagy. Siddiqui and coworkers [119] have shown that HCV infection induces phosphatase and tensin homolog (PTEN)-induced putative kinase 1 (PINK1) and Parkin on the outer surface of mitochondria to initiate mitophagy. They propose that the loss of mitochondria enhances HCV replication and suppresses cellular apoptosis. Kurt *et al.* [90] in our laboratory showed HCV infection induced expression of LAMP2A and HSC70 expression, leading to CMA. Silencing CMA pathways improved innate antiviral response and restored degradation of interferon-alpha receptor-1 expression. The autophagy response is important for persistent virus replication in cell culture. The majority of autophagy related publications verified that silencing autophagy genes by using small interfering RNAs (siRNAs), or suppression of autophagy by small molecule inhibitors, lead to inhibition of HCV replication, suggesting that autophagy is needed to continue viral RNA replication in hepatocytes.

It is likely that HCV infection induces autophagy by ER-stress dependent and independent mechanisms. Since high-level chronic ER-stress is maintained in advanced liver disease, an integrated analysis on how autophagy is induced under stressful conditions will provide useful information regarding HCV-related HCC mechanisms. Several researchers, including us, showed that HCV infection induces autophagy by ER-stress through induction of UPR and silencing the ATF6, PERK and IRE1 by siRNA, or inhibition of ER-stress by phenyl butyric acid (PBA) inhibited autophagy [53,54,57,108,115]. Inhibiting ER-stress response using small molecule or siRNA also leads to decreased HCV RNA replication and infectious HCV particle production [54,112]. Huang and coworkers claim that both HCV-N strain and JFH strain virus induce autophagy in Huh-7 cells by inhibiting Akt and mTOR pathways, supporting the hypothesis that HCV induces autophagy through nutrition depletion [114]. This study did not find any change in the AMPK pathway. Another study by the same group [108] showed that HCV core proteins induced autophagy through ER-stress by inducing PERK and ATF6 pathways. The authors showed that ATF4 and CHOP down-stream of ER-stress related to UPR signaling induced transcription of ATG12 and LC3B and autophagy. Using a persistent HCV infection model, we showed that cells treated with ER-stress inducer (TG) or mTOR inhibitor (Torin1) or HCV infection induces autophagy in Huh-7.5 cells leading to degradation of interferon-alpha receptor-1 chain of the type I IFN receptor in the lysosome [57]. Although ER-stress induced autophagy is a viable hypothesis, some researchers have showed that HCV can induce autophagy independent of ER-stress by direct interaction of viral proteins (NS5B, NS5A, p7) with cellular autophagy (ATG5, ATG12 and FIP200) and proteins that regulate autophagy [124,125,126].

It is also well recognized that autophagy enhances HCV replication and support persistent infection in cell culture models. There is a consensus agreement among HCV researchers that autophagy is important for HCV replication. To understand how inducing cellular autophagy response helps increase viral RNA replication during infection, some investigators have shown that autophagy is important for the translation of HCV [88]. Using knockdown of ATG7 in Huh 7.5 cells, one group showed that autophagy is required for efficient production of HCV particles but not for the replication of viral RNA [118]. Another group has shown that autophagy plays an important role in the creation of defective autophagosome-like structures that support HCV RNA replication [113]. HCV induces the cellular autophagy process to benefit its persistent nature of infection by manipulating host cell survival and innate antiviral pressure for successful infection. One recent study provides evidence for how persistent virus replication interacts with the cellular autophagy network that leads to defective autophagy [126]. The authors used the two-hybrid screening approach to identify autophagy proteins that interact with viral proteins and their results indicate that IRGM (immunity associated GTPase family M) protein interacts with ATG5 and ATG10 to promote HCV replication. They showed that silencing IRGM gene impaired HCV induced autophagy response and virus production. Taken together, all these results suggest that cellular autophagy response alleviates the viral induced chronic ER-stress in order to establish chronic infection in the hepatocytes. The mechanism for how HCV infection induces different types of autophagy response with regard to the activation of three branches of UPR signaling is not well understood.

## 6. Autophagy Serves a Prosurvival Role That Favors Persistent HCV Infection

**Figure 6:**
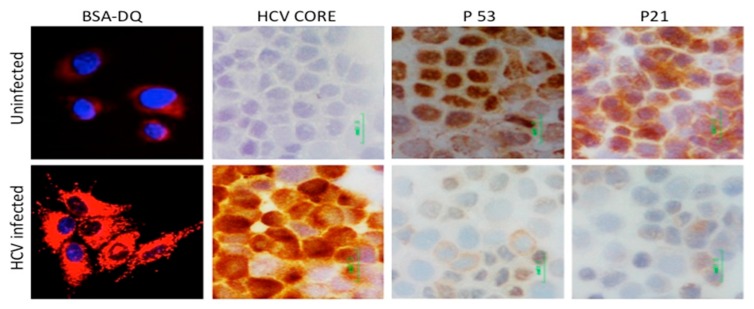
Persistent HCV infection in cell culture degrades p53 tumor suppressor and p53 target genes. Huh-7.5 cells were infected with HCV JFH1 chimera virus expressing firefly luciferase. Uninfected and infected Huh-7.5 cells on Day 21 were examined for autophagy induction by BSA-DQ staining under a fluorescence microscope. The expression of HCV core, p53 and its target gene p21 were examined by immunostaining.

Cells use two different surveillance mechanisms to block virus replication and spread. One mechanism involves the production of interferon, which directly inhibits virus replication through autocrine and paracrine mechanisms through the IFN-activated Janus kinase/signal transducers and activators of transcription (JAK/STAT) pathway. The other mechanism involves blocking the spread of infection by inducing p53-mediated cellular apoptosis [127,128,129,130,131]. Due to this blocking mechanism, many viruses, as well as bacteria, regulate p53 signaling in favor of their continued survival. The p53 protein is induced during the cellular stress response, leading to activation of its target genes involved in cellular apoptosis [132,133]. Prior studies have reported that HCV protein expression either activates [134,135] or represses p53 functions [136,137,138,139,140,141,142,143,144,145]. These studies have been conducted using HCC cell lines, which are known to have defects in p53 pathways or to express functionally defective mutant p53 [146]. We now have new evidence suggesting that HCV infection induces degradation of p53 tumor suppressors through chaperone-induced autophagy in both PHHs as well as infected Huh-7.5 cells to improve cellular surveillance of persistent infection in the host [61]. Immunocytochemical staining shows that HCV replication in Huh-7.5 cells degrades p53 tumor suppressors and p53 target genes p21 (Figure 6). As the loss of p53 function is associated with the majority of human cancers, these findings may contribute to our understanding of how the HCV-induced autophagy response favors cell survival pathway. Other researchers showed that the HCV-induced autophagy response improves cell survival through other mechanisms. A study by Taguwa and coworkers [117] showed that suppression of autophagy in HCV replicon cell culture induced severe cytoplasmic vacuolation and cell death, suggesting that autophagy improves cell survival for persistent HCV replication. Srivastava and coworkers [124] showed that HCV induced autophagy upregulated Beclin1 and activated mTOR levels through promoting cell growth. Recently, Alem Siddiqui’s group discovered that HCV induced mitochondria fission and mitophagy in cell culture [89]. They showed that inhibition of this mitophagy triggers robust apoptosis measured by increases in cytochrome C release, caspase activity, and cleavage of poly (ADP-ribose) polymerase. These evidences suggest that HCV induced autophagy responds to improve cell survival under ER-stress by multiple mechanisms. Additional investigations in this area should increase our understanding of the molecular mechanisms for how cellular adaptation to chronic ER-stress induces hepatocyte survival.

Cellular autophagy response is induced during infection with wide range of DNA and RNA viruses [146]. It has been shown that some viruses needed autophagy processes for their own replication while some other viruses inhibit the cellular autophagy process for the establishment of persistent virus infection (nicely reviewed [107]. For example, Sindbis virus, HCV, hepatitis B virus and dengue virus infection induced cellular autophagy to sustain replication. Autophagy response found to be beneficial for some viruses, such as dengue virus for generating energy through degradation of lipid [148]. Some other viruses such as Kaposi’s sarcoma herpes virus (KSHV), herpes simplex virus 1 (HSV-1), human immunodeficiency virus-1 (HIV-1), influenza A virus (FluAv), coxsackievirus B3 (CVB3) and poliovirus have been found to inhibit the cellular autophagy processes [107]. Some viruses including human herpesvirus 6, adenovirus and measles virus, induce cellular autophagy process during viral entry by direct interaction with cell surface receptors [107]. In addition to prosurvival role, autophagy induction during HCV replication is also used to suppress host antiviral innate immunity for the establishment of chronic infection.

## 7. HCV Induces Autophagy Response to Suppress Innate Antiviral Response

Induction of cellular autophagy process during virus infection has been shown to stimulate the innate immune response, including toll-like receptors (TLR) signaling and antigen presentation in complex with major histocompatibility complex (MHC)-1 and MHC-II molecules [149,150,151,152]. HCV induced autophagy has been found to play a major role in evading host microbial defense mechanisms at the level of innate and adaptive immune response [153]. In general, virus infection produces IFN due to a series of molecular interactions between viral pathogen-associated molecular patterns (PAMP) and host pattern recognition receptors (PRRs), (TLR), RIG-1-like receptors (RLR), and the nucleotide oligomerization domain (NOD)-like receptors [154,155]. In such case, hepatocytes produce IFNs after HCV infection as a first-line innate antiviral defense mechanism. The endogenously produced IFNs play important roles in inducing viral clearance and the establishment of the antiviral state through binding to cell surface receptors and activation of JAK/STAT signaling that results in induction of interferon inducible genes (ISGs). IFN-α receptor (IFNAR)-regulated genes include *IRF7*, which induces transcription of IFN-α genes, *PKR* and other ISGs to enhance viral recognition and antiviral response. This PRR-mediated antiviral signaling controls the infection. Prior studies have shown that HCV proteins (core, E2, NS3/4A, NS4B, and NS5A) employ wide varieties of mechanisms to inhibit IFN production and expression of interferon stimulated genes (ISGs), which provides an explanation why HCV develop high rate of chronic infection that escape IFN-based antiviral treatment. Many previous reviews described the mechanism for how different HCV viral proteins block the cellular innate antiviral signaling; however, as these analyses are not related to autophagy, they will not be reviewed here [156,157]. Recent studies have demonstrated that HCV induced autophagy response inhibits innate antiviral response and production of endogenous IFN. Two groups have demonstrated that HCV induced autophagy response inhibits innate antiviral responses, plus knockdown of autophagy gene increased the production of endogenous interferon [54,158]. This conclusion was also supported by results showing that inhibition of autophagy by chloroquine or bafilomycin results in increased expression of IFN, IFN-stimulated genes and innate antiviral immune response [54]. Silencing of Beclin-1 and ATG7 in HCV infected cells by siRNA inhibited autophagy and induced IFN signaling and expression of ISGs [157]. At present, there is no experimental evidence to support the conclusion that the autophagy response modulates adaptive immunity during chronic HCV infection. Emerging new information indicate that development of protective adaptive immunity against virus infection depends on the synergistic intersection between host PRR, ER-stress genes (UPR) and inflammation at the level of pro-inflammatory cytokines and type I IFN production [158]. It needs to be tested whether HCV infection leads to the inhibition of synergistic interaction between UPR, PRR could be playing a role in mounting an inadequate adaptive response.

## 8. Autophagy Response Impairs HCV Clearance by IFN-α /RBV Based Antiviral Therapy

IFN-α and RBV have been used as combination therapy for chronic HCV infection for many years prior to DAAs based antiviral treatment. Only half of patients are able to clear the virus with this combination therapy and many develop resistance to IFN-α plus RBV antiviral therapy. The mechanism for why some patients clear the virus and some do not clear the virus infection by IFN-α plus RBV combination therapy is not clear. Several years of clinical research have provided evidence that a number of viral- and host-related factors interfere with IFN-α and ribavirin treatment induced virus clearance. These include virus genotype, viral load, HIV co-infection, gender, race, obesity, insulin resistance, fibrosis, IFN-lambda3 (IFNλ3, IL28B) genotype and pre-activation of IFN-inducible genes and others. IFNs are divided into three types based on the nature of signaling they induce after binding to the cell surface: Type I IFN, Type II IFN and Type III IFN. Type I IFNs bind to the IFNAR complex consisting of two major subunits: IFNAR1 and IFNAR2. Type II IFNs are referred to as interferon gamma (IFNγ) and are produced primarily by T lymphocytes and natural killer cells. IFNγ binds to a distinct cell surface receptor called the interferon gamma receptor (IFNGR) consisting of two subunits, IFNGR1 and IFNGR2. Type III IFNs are referred to as interferon-lambda (IFNλ). They are also known as IL28A (IFNλ2), IL28B (IFNλ3) and IL29 (IFNλ1), respectively. Type III IFNs signal through JAK/STAT signaling and induce an antiviral state similar to type I IFN [159]. Many investigators, including our laboratory, have shown that IFN-α effectively inhibits HCV replication in cell culture models [57,160,161]. A series of publications from our laboratory as well as other laboratories have verified that the JAK/STAT pathway induced by interferon-alpha is critical for the antiviral mechanism against HCV in cell culture models [162,163,164,165,166]. All three types of IFNs (type I, type II and type III) have been shown to inhibit HCV replication in cell culture by targeting the 5’-UTR of HCV RNA genome used for IRES mediated translation [162]. The combination of IFN-α and RBV treatment synergistically inhibits HCV IRES translation through prevention of polyribosome formation using two different mechanisms involving PKR activation and depletion of intracellular guanosine pool through inhibition of inosine-5′-monophosphate dehydrogenase (IMPDH) [165].

We showed that HCV induced autophagy response impairs HCV clearance by exogenous IFN-α but not by IFNλ [57]. The explanation for this is that HCV replication induced autophagy response and impaired the expression of IFNAR1 chain of the type I IFN-receptor, leading to defective JAK/STAT signaling. The HCV-induced autophagy response did not alter the expression of type III IFN receptors, which is why IL29 induced HCV clearance. These results are consistent with a previous report that showed vesicular stomatitis virus (VSV) and hepatitis C virus infection induce IFNAR1 degradation through ER-stress mechanism [167]. To understand why HCV induced autophagy response selectively target IFNAR1 but not the Type III IFN-receptors, we examined the mechanisms of IFNAR1 degradation in HCV cell culture model. We examined whether selective degradation of IFNAR1 could involve CMA. CMA is a type of autophagy responsible for selective degradation of cytosolic protein bearing certain consensus amino acid motif (KFERQ); the targeting sequence is found to be glutamine (Q) flanked at either end by a hydrophobic (F, I, L, V) an acidic (E, D), a basic (R, K) and the second hydrophobic or basic amino acid [168,169,170]. Any cytosolic protein containing this motif is recognized by HSC70, a cytosolic chaperone protein called the heat-shock cognate protein. This protein complex then translocates to the lysosome surface where the complex binds to CMA receptor LAMP2A. The protein complex then translocates to the lysosome where the CMA substrates are rapidly degraded. We found that there is a CMA consensus amino acid motif (QKVEV) present at amino acid 34 in the human IFNAR1 protein and that this motif is absent in the IFNλ receptor. Activators of chaperone-mediated autophagy, including 6-aminonicotinamide and nutrient starvation, decreased IFNAR1 levels in Huh-7.5 cells. Co-immunoprecipitation, colocalization and siRNA knockdown experiments revealed that IFNAR1 but not IFNλ receptor 1 (IFNLR1) interacts with HSC70 and LAMP2A, which are core components of chaperone-mediated autophagy (CMA) [90]. These studies provide an explanation why stress response selectively targets IFNAR1 for degradation.

**Figure 7:**
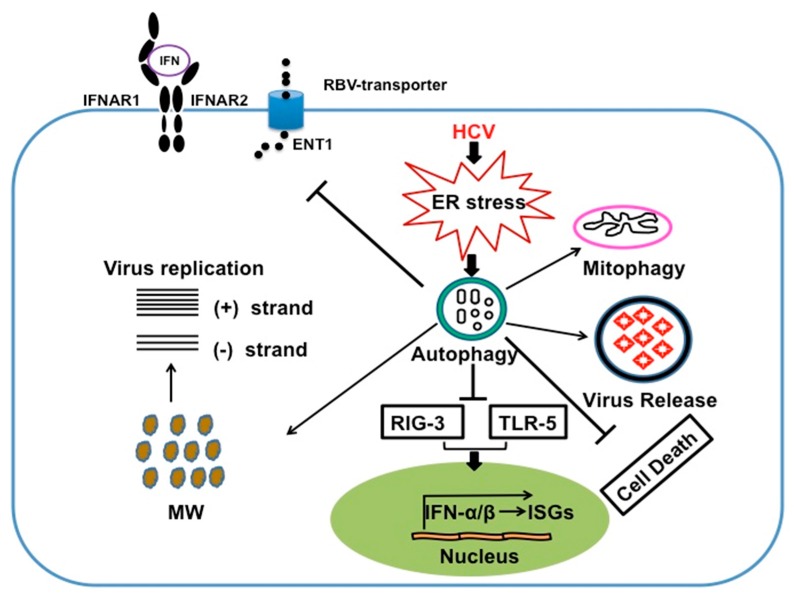
Diagram illustrating how autophagy response adapts to HCV induced cellular stress response leading to chronic infection. HCV replication induces ER-stress/UPR response that triggers autophagy that favors cell survival through inhibition of apoptosis and mitophagy. HCV induced autophagy response inhibits interferon production and interferon inducible genes (ISG) transcription and impairs Janus kinase/signal transducers and activators of transcription (JAK/STAT)signaling by degradation of IFN-α receptor 1 (IFNAR1) and RBV transporter. Autophagy response created autophagosome that support HCV replication and release.

RBV remains an important component of interferon-free hepatitis C treatment regimens. The reason why RBV alone does not inhibit HCV replication effectively has not been established. We found that HCV replication in persistently infected cultures induces an autophagy response that impairs RBV uptake by preventing the expression of equilibrative nucleoside transporter 1 (ENT1). The Huh-7.5 cell line treated with an autophagy inducer, Torin 1, downregulated membrane expression of ENT1 and terminated RBV uptake. In contrast, the autophagy inhibitors hydroxychloroquine (HCQ), 3-methyladenine (3-MA), and bafilomycin A1 (BafA1) prevented ENT1 degradation and enhanced RBV antiviral activity. The HCV-induced autophagy response, as well as treatment with Torin 1, degrades clathrin heavy chain expression in a hepatoma cell line. Reduced expression of the clathrin heavy chain by HCV prevents ENT1 recycling to the plasma membrane and forces ENT1 to the lysosome for degradation. This study provides a potential mechanism for why RBV antiviral activity is impaired in persistently HCV-infected cell cultures and suggests that inhibition of the HCV-induced autophagy response could be used as a strategy to increase RBV antiviral activity against HCV infection. We also showed that the HCV-induced autophagy response also impairs the hepatocyte surface expression of ENT1 through degradation of clathrin-heavy chain molecules [116] Inhibition of the autophagy response by siRNA mediated silencing of ATG5 or treatment with hydroxychloroquine improved HCV clearance by IFN-α and ribavirin [57,116]. These two studies provide a potential mechanism how HCV induced autophagy response impairs response to antiviral therapy using IFN-α and RBV combination. Liver cirrhosis is another host-related factor affecting the success of IFN-α and RBV combination therapy of chronic HCV infection. We verified whether the increased ER stress and autophagy response are related to impaired expression of type I and type II IFN signaling in HCV-infected human liver tissue [60]. Our results show ER stress and autophagy marker expression are induced in HCV-infected primary human hepatocytes and liver tissues of patients with chronic liver disease (CLD). Compared to normal human liver tissues, ER stress and autophagy response are increased during CLD, but IFNAR1 expression is significantly reduced in HCV-induced CLD with or without cirrhosis. These data verify the results of cell culture studies indicating impaired type I IFN signaling during CLD and provide an explanation for the reduced viral clearance in cirrhotic patients receiving IFN-α and RBV combination therapy. In summary, HCV induced autophagy process to benefit its persistence nature of infection by manipulating host cell survival and innate antiviral pressure for successful infection. Increased autophagy response plays an important role in inducing viral clearance by IFN/RBV antiviral therapy (Figure 7).

## 9. Interplay among UPR, Autophagy, Inflammation and Evolution of Chronic Liver Disease

A number of researchers have now verified interplay among ER-stress, autophagy and HCV infection *in vitro*. The role of autophagy in the development of progressive liver disease is still speculative. The molecular details for how ER-stress induces such autophagy response that triggers liver disease progression will be an active area of future investigation. We discuss below the molecular interaction between UPR, autophagy that plays a critical role in cell apoptosis, cell survival, chronic infection and carcinogenesis. Mechanisms of acute and chronic liver injury in humans have been linked mainly to infection by hepatitis viruses, alcohol abuse, and excess fat accumulation. In some individuals, combinations of all of these agents contribute to liver disease progression. All these viral and non-viral agents target mostly hepatocytes, the predominant cell type in the liver that is subject to acute and chronic injury. One of the host-related factors responsible for the progression of liver disease is the degree of hepatocellular injury. For these reasons, an increase in serum aminotransferase has been used by physicians as one of the surrogate markers for assessing the extent of hepatic injury in patients with chronic liver diseases of both viral and non-viral etiology.

The liver injury is caused by three distinct types of cell death: apoptosis, necrosis, and autophagy. Apoptosis is a form of cell death mediated by an intracellular proteolytic cascade, which results in non-traumatic cell death. Apoptotic cells are usually phagocytosed either by neighboring cells or by a macrophage. Necrosis is a form of cell death mediated by acute injury in which the cell swells, bursts, and spills its contents into surrounding areas, causing an inflammatory response. Autophagy is an intracellular degradation process required for maintaining cellular homeostasis. Hepatocytes infected with HCV can compensate for the mild to moderate ER-stress by UPR signaling, but excessive ER-stress can lead to cellular apoptosis. For example, HCV infection induces the autophagy response to degrade organelles and long-lived proteins needed to generate energy and sustain virus replication in hepatocytes. If uncontrolled, the induced autophagy response could lead to autophagic cell death and the elimination of infected hepatocytes in the liver. Available evidence suggests that the autophagy response is deregulated in chronic liver disease and liver cirrhosis, which can lead to HCC. Therefore, in the following section we will discuss the possibilities that autophagy play as a prodeath (tumor suppressor) or prosurvival (oncogenic) during chronic liver disease induced by HCV infection.

Autophagy acts as a tumor suppressor in chronic liver disease. Apoptosis and autophagy are interrelated biological processes important for maintaining tissue homeostasis and carcinogenesis. It has been shown that the genes controlling apoptosis (Bcl-2) are involved in carcinogenesis. Oncogenic mutations in the Bcl-2 gene that inhibit apoptosis can lead to tumor initiation, progression, or metastasis. Alternatively, oncogenes can cause inhibition of apoptosis leading to multistage carcinogenesis. A number of excellent reviews have described the role of hepatocyte apoptosis in prodeath (tumor suppression) during chronic liver injury and carcinogenesis [171,172]. Likewise, hepatocellular autophagy can play a tumor suppression role in chronic liver disease, and an impaired cellular autophagy response due to mutation in Beclin/Bcl-2 gene or inhibition of cellular apoptosis by HCV can lead to malignant transformation and hepatocellular carcinoma.

**Figure 8:**
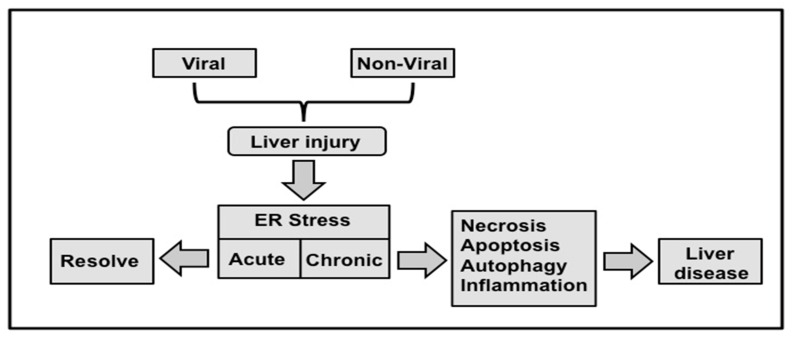
Diagram showing the relationship among ER-stress, autophagy, and inflammation in the progression of liver disease induced by HCV infection. The ER-stress can be tolerated from days to years through autophagy induction. Even if apoptotic cell death occurs, some extend due to the ER-stress but the majority of cells survive due to autophagy during HCV infection. UPR can induce autophagy and inflammation, which contribute to liver disease progression.

Autophagy also plays a prosurvival/oncogenic role during chronic HCV infection. The high-level expression of UPR genes plus autophagy in chronic liver disease, liver cirrhosis and HCC suggests that chronic ER-stress persists in the advanced stages of several liver diseases [60]. This finding from human studies suggests that the majority of HCV infected hepatocytes can tolerate chronic ER-stress response for days to years with minimal cell death. These results clearly support the prosurvival role of UPR and autophagy mechanism proposed initially by the Kaufman group (Figure 8) for how the UPR allows adaptation of chronic ER-stress that improves the survival of hepatocytes instead of inducing apoptosis [173]. The autophagy is induced to improve hepatocyte survival under ER-stress, but the pathological implication of maintaining elevated autophagy levels in the evolution of chronic liver disease in humans is unknown. To date, a consistent autophagic tumor suppressor mechanism causing the development of chronic liver disease, cirrhosis, and HCC has not been identified. We propose that additional research is needed to determine why the interplay of chronic ER-stress, autophagy and cell survival pathways lead to liver cirrhosis and HCC induced by chronic HCV infection Understanding the mechanisms of UPR and autophagy regulation during the progression of chronic liver disease is highly significant since defective autophagy response has been linked to cancer development [102].

The pathogenesis of chronic liver disease is also contributed by host immune response and inflammation. The molecular details of how HCV persists in the infected host by overcoming innate, adaptive immunity and escaping apoptotic cell death are also not well understood. HCV subversion of cellular stress response appears to be a key event that explains the mechanisms of viral persistence and viral carcinogenesis. The relationship between ER-stress, innate immunity and adaptive immunity is emerging (reviewed [38,174,175]). Available literature indicates that the mechanism of HCC development by HCV infection can be attributed to a combination of direct (virus-related) and indirect (host-related) inflammatory responses [176,177]. There is substantial evidence that host-related impaired immune protection contributes to HCC development [178,179,180,181]. The molecular details of how HCV persists in the infected host by overcoming innate, adaptive immunity and escaping apoptotic cell death are also not well understood.

The innate immune response generated during virus infection is mediated by various cell types (monocytes, macrophages, dendritic cells), PRR and inflammatory cytokines that play an important role in viral clearance. It is not clear how UPR interacts with innate immune response to initiate chronic inflammation. Recent studies show that the synergistic interaction of UPR and PRR results in the production of pro-inflammatory cytokines (TNF-α, IL-6, IL-1b and IL-23), type I IFN [38,158]. The UPR activates the inflammatory cytokine transcription and enhances cytokine production in response to viral cytosolic PRR engagement (reviewed nicely [158]). These studies indicate that UPR controls recruitment and activation of adaptive immune cells including T and B cells, by these cytokines and MHC antigen expression that results in inflammation and elimination of infected cells. At present, our understanding on the ER-stress, UPR and innate and adaptive immune response is limited and beyond the scope of this review. Future research on this emerging hot topic will increase our understanding for how the crosstalk between cellular stress and immune regulation in chronic liver disease is induced by HCV. Overall, the molecular mechanisms by which HCV develops persistent infection in the hepatocytes by maintaining a delicate balance between viral induced stress response and cell survival is not well understood. This is an important area of research that may increase our understanding of the interplay between long-lasting chronic HCV infection and development of hepatocellular cancer.

## 10. Summary and Conclusions

Hepatitis C virus is a major human pathogen associated with development of severe liver disease and hepatocellular carcinoma. The molecular details of how HCV infection leads to high rates of chronic infection are unknown. As described in this review, ER-stress and UPR induced during chronic liver disease can be related to HCV, HBV and other non-viral causes. The autophagy response is clearly needed for persistent HCV replication. We also discuss in this review how autophagy induction favors the establishment of persistent HCV infection by inhibiting innate antiviral signaling and improving cell survival. Although HCV induces ER-stress and autophagy, their cause–effect relationship is not clear. Therefore, future research should elucidate the mechanisms of autophagy initiation related to ER-stress/UPR in HCV infection. Most of the information related to HCV induced ER-stress and autophagy is now derived from cell culture studies, therefore verification of these findings *in vivo* should allow better understanding of their role in the evolution of liver disease.
